# Development of a Sensor System for the Determination of Sanitary Quality of Grapes

**DOI:** 10.3390/s130404571

**Published:** 2013-04-08

**Authors:** Chiara Zanardi, Luca Ferrari, Barbara Zanfrognini, Laura Pigani, Fabio Terzi, Stefano Cattini, Luigi Rovati, Renato Seeber

**Affiliations:** 1 Department of Chemical and Geological Sciences, University of Modena and Reggio Emilia, via G. Campi 183, 41125 Modena, Italy; E-Mails: barbara.zanfrognini@unimore.it (B.Z.); laura.pigani@unimore.it (L.P.); fabio.terzi@unimore.it (F.T.); seeber@unimore.it (R.S.); 2 Department of Engineering “Enzo Ferrari”, University of Modena and Reggio Emilia, strada Vignolese 905, 41125 Modena, Italy; E-Mails: luca.ferrari@unimore.it (L.F.); stefano.cattini@unimore.it (S.C.); luigi.rovati@unimore.it (L.R.)

**Keywords:** amperometric sensor, glycerol, glycerol dehydrogenase, diaphorase, *Botrytis cinerea*, screen printed electrodes, micropumps, microprocessor, grapes

## Abstract

An instrument for the automatic quantification of glycerol in grapes has been developed. We verify here that this analyte can be used as a benchmark of a serious disease affecting the grapevines, namely *Botrytis cinerea*. The core of the instrument is an amperometric biosensor consisting of a disposable screen printed electrode, generating the analytical signal thanks to a bi-enzymatic process involving glycerol dehydrogenase and diaphorase. The full automation of the analysis is realised by three micropumps and a microprocessor under control of a personal computer. The pumps allow the correct and constant dilution of the grape juice with a buffer solution also containing [Fe(CN)_6_]^3−^ redox mediator and the injection of NAD^+^ cofactor when the baseline signal reaches a steady state; the instrument leads to automated reading of the analytical signal and the consequent data treatment. Although the analytical method is based on an amperometric technique that, owing to heavy matrix effects, usually requires an internal calibration, the analyses indicate that a unique external calibration is suitable for giving accurate responses for any grapes, both white and black ones.

## 1. Introduction

The production of wine of good quality is closely related to the sanitary status of the original grapes. Great attention is nowadays directed toward *Botrytis cinerea*, a fungal disease responsible for significant alterations of the chemical composition of grapes. Although this infection can be also driven to “noble rot”, used for the production of special wines such as Passito, Tokai and Amarone [1–3], in most cases it leads to “grey rot”, a serious alteration of grape integrity which negatively affects the winemaking process [4]. Skin contraction and dehydration of grapes are evident markers of the occurrence of the disease, followed by evident colour changes induced by the increased activity of enzymes such as laccase and tyrosinase; these enzymes are also responsible for the production of high levels of glycerol in the berries, *i.e.*, before must fermentation in the vats. *Botrytis cinerea* can finally induce disruption of the external skin of the berries, with consequent proliferation of acetic acid bacteria (*Acetobacter* and *Gluconobacter*) and formation of high levels of gluconic and acetic acids. These undesired fermentation processes affect the taste of the wine finally produced. For this reason, the sanitary quality of the grapes has to be very carefully evaluated before any processing. Due to the lack of portable instruments capable of making quantitative estimations directly on the field and to the rather short times available when receiving the grapes in the wine cellar, the evaluation is nowadays made by visual criteria that suffer from individual bias: the possibility of using more objective and even quantitative criteria appears definitely preferable.

Among the different chemical species produced by *Botrytis cinerea*, our attention was directed to the determination of glycerol. This molecule is routinely analysed either by a liquid chromatographic method, constituting the official method of analysis [5], or by spectrophotometric assessment of the effect of an enzymatic reaction (enzymatic kit) [6,7]. However, both these methods require the presence of qualified personnel carrying out the analysis in a suitable laboratory and are not compatible with the times required by the analysis during the reception of grapes. Moreover, the use of the enzymatic kit is also quite expensive because it requires the addition of three enzymes (glycerol kinase, pyruvate kinase and lactate dehydrogenase), two co-substrates, namely adenosine tri-phosphate (ATP) and phosphoenolpyruvate, and the coenzyme (NADH) for each sample under analysis. On the contrary, amperometric biosensors are acknowledged to be simple to use and able to very rapidly and selectively quantify the target analyte by stably fixing a very low amount of enzyme on the electrode surface; moreover, the relevant instrumentation can be also made portable. When using screen printed electrodes (SPE), the sensor results very cheap, thus even disposable, which constitutes an advantage in order to overcome the problem of the lifetime of enzymes when fixed on conducting substrates.

Different enzymes were used for the construction of amperometric biosensors for glycerol determination, including glycerol dehydrogenase (GDH) [8–12], glycerol kinase/glycerol-3-phosphate oxidase [10,12–14], pyrroloquinoline quinone (PQQ)-dependent glycerol dehydrogenase [15–17], glycerol kinase/creatine kinase/creatinase/sarcosine oxidase/peroxidase [18], glycerol kinase/pyruvate kinase/pyruvate oxidase [19] and glycerol oxidase [20]. Among the others, GDH is commercially available and, in the presence of Nicotinamide Adenine Dinucleotide (NAD^+^), leads to the formation of the redox active NADH cofactor, according with the reaction:

(1) $$\text{glycerol}+{\text{NAD}}^{+}\to \text{dihydroxyacetone}+\text{NADH}+{\text{H}}^{+}$$

Due to the high overpotential affecting the oxidation of the NADH and the severe passivation of the electrode surface, redox mediators are generally added to the electrochemical system in order to catalyse the oxidation of NADH [21]:

(2) $$\text{NADH}+{\text{M}}^{\text{ox}}\to {\text{NAD}}^{+}+{\text{M}}^{\text{red}}$$

The analytical data finally consist of the current values registered when the reduced form of the redox mediator produced by the enzymatic reaction is newly oxidised at the electrode surface. Reaction (2) can be in turn catalysed by diaphorase (DP) that can be also added to the catalytic system; in this case, it is also anchored at the electrode surface [10,12].

Amperometric biosensors consisting of GDH/DP bi-enzymatic system have been already reported in the literature for the quantification of glycerol in wines [12,22]. Since this analyte is massively produced during alcoholic fermentation, its concentration is particularly high in this matrix (from 4 to 20 g/L [12]) and high dilutions of the sample are necessary; thus, matrix effects are not particularly meaningful and the analysis can be finally carried out through an external calibration registered in a simple buffered solution [12].

To the best of our knowledge, no attempts have been made to determine the concentration of glycerol in grapes by means of amperometric biosensors. In this matrix the concentration of this chemical species is significantly lower (generally <1 g/L) with respect to wine samples, thus requiring much lower dilution factors. Moreover, the chemical composition of the sample is very different from the winery product at the end of alcoholic fermentation process.

In this paper we report the development of a fully automated instrument for the determination of glycerol in grapes. The analysis of samples coming from the same vine but containing increasing amount of grapes affected by *Botrytis* allowed us to verify that this analyte can be considered a good benchmark of the sanitary quality of the grapes. Due to the proposed instrumental setup, the analysis can be very easily carried out in a rather short time by unqualified personnel and, when required, directly on the vineyard. The core of the measuring system is an amperometric biosensor consisting of a disposable screen printed electrode on which GDH and DP are stably fixed. The oxidation of NADH formed by the enzymatic reaction is catalysed by [Fe(CN)_6_]^3−^ redox mediator, dissolved in the electrolyte solution in order to avoid its leaching from the electrode surface when working in a thin layer cell at fairly high flow rates. The automation of the system is possible thanks to three micropumps controlled by a microprocessor finally connected to a netbook.

## 2. Experimental

### 2.1. Chemicals

Enzymes glycerol dehydrogenase (GDH, E.C. 1.1.1.6, 112 U/mg) from *Cellulomonas* sp. and diaphorase (DP, E.C. 1.8.1.4, 4 U/mg) from *Clostridium kluyveri* sp. were acquired from Sigma (Milan, Italy) and used without further purification. Enzymatic kits for the spectrophotometric quantification of glycerol in grapes were acquired from Boehringer Mannheim (Milan, Italy). All the other reactants were acquired from Sigma Aldrich (Milan, Italy) and used as received.

### 2.2. Instrumentation

Electrochemical measurements have been carried out with a PalmSens handheld potentiostat/galvanostat (Utrecht, The Netherlands) interfaced with a personal computer. SPEs were acquired from EcoBioServices (Florence, Itay) and consisted of a 3 mm diameter graphite working electrode, an Ag pseudo-reference electrode and a graphite auxiliary electrode. Before use, SPEs were polarised at +1.7 V for 180 s in a 0.1 M phosphate buffer solution (PBS), 0.1 M KCl. The working electrode was then modified by a two-step procedure: in a first step 1% glutaraldehyde solution (2 μL) was spread onto the surface and let dry for 2 hours. Secondly, 1.4 μL of PBS, 2.8 UI of GDH, 0.8 UI of DP and 1.4 μL of 1% neutralized Nafion^®^ solution were carefully mixed, deposited onto the electrode surface and let dry overnight.

SPEs were inserted into a flow cell, acquired from EcoBioServices, working either under reflux conditions or in a frame of a configuration similar to that of flow injection analysis. In the first case, the solution was fluxed through the electrochemical cell at 3.5 mL/min thanks to a micropump (Bartels Mikrotechnik, Dortmund, Germany), while the additions of known volumes of sample and reactants were performed in an external vial, using micropipettes from Gilson. In the second case, two micropumps (Bartels Mikrotechnik) act on the fluxes of the electrolyte solution, also containing [Fe(CN)_6_]^3−^ redox mediator, and the grape juice, prepared as described in the next paragraph. By suitably modulating the pump flows, they automatically lead to the desired dilution of the sample and to a final flux speed of 3 mL/min. A third micropump (Bartels Mikrotechnik), inserted close to the measuring cell entrance, is activated for 2 s when the background current reaches a constant value; it allows the injection of a fixed amount of NAD^+^ into the instrumental setup. Finally, a pre-mixing chamber is inserted between NAD^+^ injection and the electrochemical cell, in order to homogenise the solution before the occurrence of the enzymatic reaction.

### 2.3. Analysis of Real Samples

All grapes were processed before the analysis, in order to make them suitable to be analysed in the instrumental device previously described. To this aim, they were minced as received and filtered off with a qualitative filtering paper and with subsequent 10 μm and 2.7 μm syringe filters. The samples were stored in the refrigerator in order to avoid any fermentation process which should alter the content of glycerol in the sample. For the analysis, all the samples were diluted in 1 to 8 ratio with the electrolyte solution, consisting of 0.05 M PBS at pH = 8.5, 0.1 M KCl and 3 mM [Fe(CN)_6_]^3−^.

## 3. Results and Discussion

### 3.1. Preliminary Analyses in Reflux Regime

Preliminary tests have been carried out in the absence of analyte, in order to test the conductivity of the electrode when coated by a poorly conductive layer containing the enzymes of interest. For this purpose, cyclic voltammetric curves have been registered in the electrolyte solution containing [Fe(CN)_6_]^3−^ before and after the deposition of the biomolecules. In both cases, a peak-to-peak separation of *ca.* 200 mV was computed, indicating that the electrode coating is permeable to chemical species present in the solution. On the basis of such a voltammetric test, we decided to perform amperometric tests in the presence of the analyte, at the potential value of +0.35 V.

A first set of measurements have been carried out with grapes of good quality, *i.e.*, not affected by *Botrytis*. In order to evidence a possible matrix effect, calibration plots have been registered by standard additions of glycerol in solutions containing different amount of grape juice. A linear plot was obtained: the line slope is strongly related to the amount of grape juice in the electrolyte solution, being the lower the higher the quantity of added sample. This aspect indicates the occurrence of a matrix effect which does not allow for the use of an external calibration plot registered in pure buffer solution. On the other hand, amperometric measurements are very commonly affected by the composition of the matrix, so that internal calibration plots are generally preferred.

The same analysis also evidences that the higher the amount of grape juice initially added to the electrolyte solution containing [Fe(CN)_6_]^3−^ the higher the line intercept of the calibration plot. Since the concentration of glycerol in the sample is rather low (0.03 gL^−1^), these current values are ascribable to interfering chemical species directly oxidised by the redox mediator; this conclusion is also supported by a further amperometric measurement which evidences a direct correlation between the oxidation current registered in the absence of NAD^+^ and the added amount of grape juice. To eliminate this interference, we decided to use the difference between the current value registered in the presence of cofactor (i_NAD_) and before this addition (i_FeCN_) as the analytical signal.

**Figure 1.**
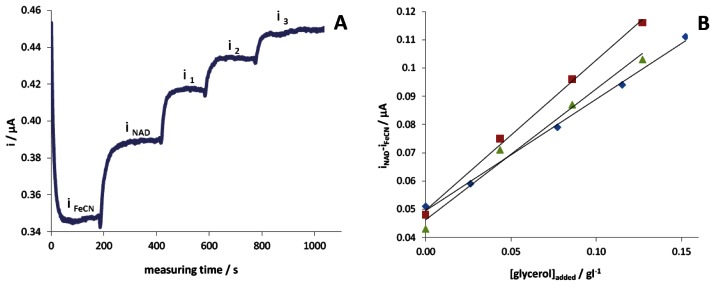
(**A**) Amperometric signal obtained at +0.35 V by subsequent additions of glycerol standard solution in a grape juice diluted eight times with the buffer solution containing [Fe(CN)_6_]^3−^; (**B**) three consecutive calibration plots obtained from the analysis of the same sample.

By following this approach, a sample of grapes affected by *Botrytis* has been analysed three times under reflux regime, by standard additions method (see Figure 1) and the calculated mean value has been compared with the one determined by enzymatic kit. It was thus possible to verify that the sensor, working in these experimental conditions, is able to determine the concentration of glycerol in grape juices with good accuracy and with 16% relative standard deviation.

However, as it is well evident from Figure 1(A), the time required to perform the analysis under these conditions is too long and the standard additions cannot be very simply automated.

Three grape juices coming from different vines (white and black grapes have been considered) have been randomly analysed four times each, under experimental conditions similar to those reported in Figure 1. These analyses demonstrate that the calibration slopes are not significantly different, thus resulting independent of the origin of the grapes. This aspect permits us to speed up the analysis, by exploiting an external calibration plot, carried out in a sample prepared from healthy grape at the beginning of the analysis set. The analyses demonstrated that the calibration plot can be used for an entire day of measurements carried out with the same electrode. This finding significantly reduces the dead times of the analysis, allowing the use of the sensor over a whole work day.

### 3.2. Analyses with the Automated Analytical Instrument

**Figure 2.**
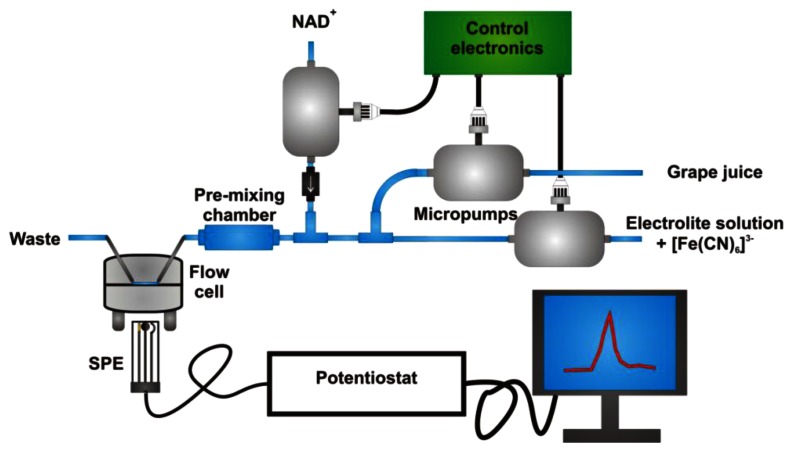
Scheme of the portable and automated instrument for the analysis of glycerol.

The final instrumental system, assembled with the aim to make the analysis completely automated, is schematised in Figure 2. Two micropumps, set at different speed, allow the desired dilution of the grape juice with the buffer solution containing the redox mediator. Spectrophotometric tests performed by adding bromothymol blue to the grape juice during an entire set of measurements allowed us to verify that a constant dilution factor was maintained.

**Figure 3.**
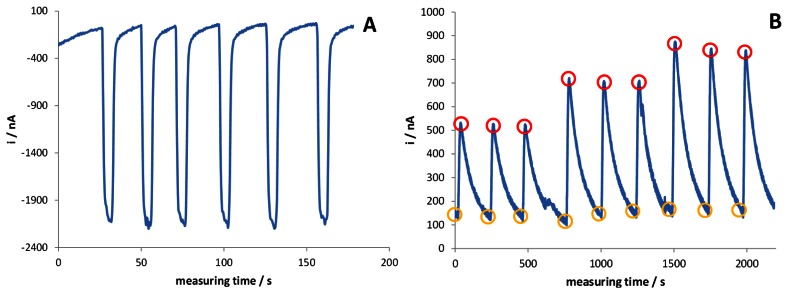
Amperometric measurements of (**A**) six subsequent injection of [Fe(CN)_6_]^3−^ through the NAD^+^ micropump in a flux of 0.05 M PBS and 0.1 M KCl (E = −0.2 V); (**B**) NAD^+^ injections in flux containing glycerol (0.11, 0.19, 0.25 gL^−1^ – 3 replicates for each sample) suitably diluted with the buffer solution, also containing [Fe(CN)_6_]^3−^, accordingly with the dilution factor due to the micropumps; red circles are for i_NAD_, yellow circles for i_FeCN_ (E = +0.35 V).

A third micropump, activated when the current baseline reaches a steady state, introduces the desired quantity of cofactor into the system; a pre-mixing chamber was inserted into the line just before the entrance to the electrochemical cell, in order to homogenise NAD^+^ to the solution under test. The good reproducibility of the cofactor injection into the electrochemical cell was verified by replacing NAD^+^ with [Fe(CN)_6_]^3−^ redox mediator and registering the current peak after its addition, performed by the micropump, to a solution containing only the supporting electrolyte. Quite obviously, in this case the applied potential was set at −0.2 V to register [Fe(CN)_6_]^3−^ reduction to [Fe(CN)_6_]^4−^, when the redox mediator is injected into the system for a fixed interval time [see Figure 3(A)]. This test also demonstrates that the time required to eliminate the reactant from the electrochemical cell, *i.e.*, to register a signal not significantly different from the original baseline, is *ca.* 25 s; this aspect indicates that the analysis is possible in a very short time.

Figure 3(B) reports the amperometric traces recorded when NAD^+^ is added to standard solutions of glycerol suitably diluted with the electrolyte solution containing [Fe(CN)_6_]^3−^ redox mediator. Three different analyte concentrations are reported here. As in the previous case, the analytical signal finally consists of the difference between the current peak value measured after the injection of NAD^+^ (i_NAD_) and that registered in the steady state regime, just before the addition of the cofactor (i_FeCN_). It can be noticed that the instrumental setup gives highly reproducible signals, proportional to the concentration of glycerol, with a mean relative standard deviation of 4%. However, in this case, the recovery time is significantly longer (*ca.* 100 s), suggesting that a portion of the injected NAD^+^ is adsorbed at the electrode surface, the desorption being rather slow.

**Figure 4.**
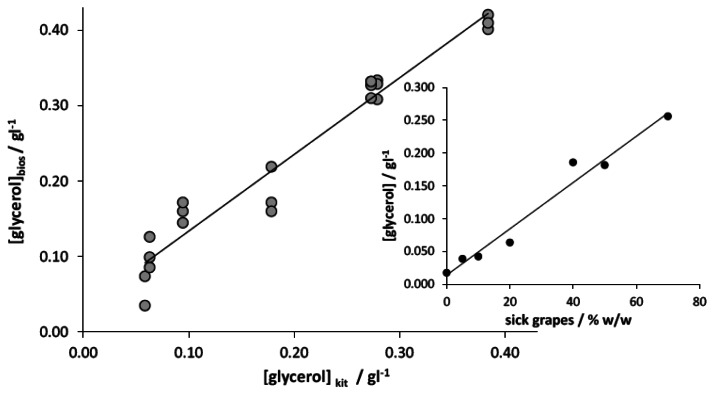
Correlation plot between the concentration of glycerol estimated with the developed automated instrument and with the enzymatic kit; samples of grapes from the same vine have been considered. Confidence limits (α = 0.05) for slope and intercept are within brackets; r^2^ = 0.95. Inset reports the correlation between the glycerol concentrations estimated with the enzymatic kit and the amount of grapes affected by *Botrytis*.

The automated instrument was then tested with grape juices. In order to concurrently verify the efficiency of the glycerol content for a correct evaluation of the entity of *Botrytis* disease, the samples consisted of grapes coming from the same vine but with different amounts of sick bunches. The correlation between the glycerol concentration, estimated with the enzymatic kit, and the amount of grapes affected by *Botrytis* is reported in the inset of Figure 4; this analysis demonstrates the actual effectiveness of the parameter chosen for the correct classification of the grapes based on the relevant sanitary level.

The quantification of glycerol with the developed amperometric instrumental setup has been carried out on the basis of an external calibration plot; it was calculated by analysing seven standard solutions prepared starting from the healthy grape and performing three replicates for each standard solution. Figure 4 shows the good correlation between the concentration values determined by the developed instrument, with respect to those estimated by the enzymatic kit. As observed, the regression slope, not significantly different from 1 (α = 0.05), and the intercept, not significantly different from 0 (α = 0.05), indicate the high accuracy of the measures obtained with the proposed instrument. Moreover, by restricting the analysis to only one measure for each sample, the glycerol quantification can be performed in a time not longer than 3 min.

**Figure 5.**
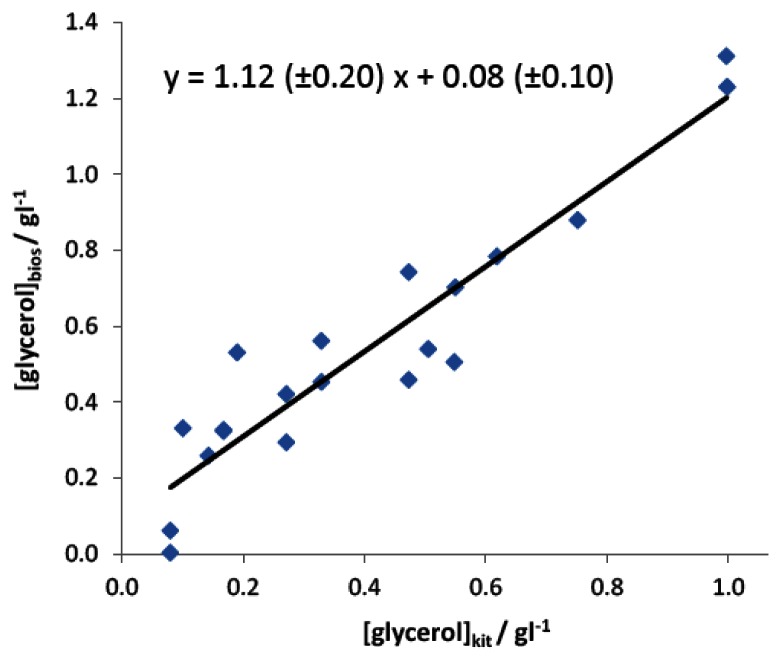
Correlation plot between the concentration of glycerol estimated with the enzymatic kit and the developed automated instrument; grapes of different variety and geographic origin have been reported. Confidence limits (α = 0.05) for slope and intercept are within brackets; r^2^ = 0.88.

Finally, the instrument was tested with a wider number of samples coming from different vines, both considering white and red grapes, as well as different geographic origins. As in the previous case, the quantification of glycerol in these samples has been carried out on the basis of an external calibration plot computed on healthy grapes. Figure 5 reports the correlation between the values calculated by the developed analytical device with respect to those coming from the reference spectrophotometric method.

As observed, the regression slope, not significantly different from 1 (α = 0.05), and the intercept, not significantly different from 0 (α = 0.05), indicate that the instrument gives an accurate determination of glycerol content in grape samples. However a higher standard deviation was calculated in this case, which badly affects both the confidence limits of slope and intercept and the correlation coefficient. This result obviously derives from the necessary use of a sample of grape juice for the determination of regression line different from those where analyses were finally carried out.

## 4. Conclusions

After a rapid and simple sample pre-treatment, the developed instrument is able to automatically quantify the amount of glycerol contained in grapes. Despite the fact that accuracy is affected by trivial errors induced by the automation and by the use of an external calibration in a similar, though different matrix, the instrument can correctly classify grapes, on the basis of the content of glycerol. As a matter of fact, we have proven that this chemical species is a suitable benchmark for the classification of grapes according to their relevant sanitary level. The proposed instrument is portable, simple to use by non-qualified personnel, and cheap; moreover, when limiting the analysis to only one measure, the time required to quantify glycerol in the samples is reduced to less than 3 minutes.
